# Author Correction: Cohort-wide deep whole genome sequencing and the allelic architecture of complex traits

**DOI:** 10.1038/s41467-018-07730-9

**Published:** 2018-12-19

**Authors:** Arthur Gilly, Daniel Suveges, Karoline Kuchenbaecker, Martin Pollard, Lorraine Southam, Konstantinos Hatzikotoulas, Aliki-Eleni Farmaki, Thea Bjornland, Ryan Waples, Emil V. R. Appel, Elisabetta Casalone, Giorgio Melloni, Britt Kilian, Nigel W. Rayner, Ioanna Ntalla, Kousik Kundu, Klaudia Walter, John Danesh, Adam Butterworth, Inês Barroso, Emmanouil Tsafantakis, George Dedoussis, Ida Moltke, Eleftheria Zeggini

**Affiliations:** 1 Department of Human Genetics, Wellcome Sanger Institute, Hinxton, CB10 1SA United Kingdom; 2 Division of Psychiatry, University College of London, London, W1T 7NF United Kingdom; 3 UCL Genetics Institute, University College London, London, WC1E 6BT United Kingdom; 4 Department of Medicine, Addenbrooke’s Hospital, University of Cambridge, Hills Road, Cambridge, CB2 0QQ United Kingdom; 5 Wellcome Centre for Human Genetics, University of Oxford, Oxford, OX3 7BN United Kingdom; 6 Institute of Translational Genomics, Helmholtz Zentrum München–German Research Center for Environmental Health, Neuherberg D-85764, Germany; 7 Department of Health Sciences, College of Life Sciences, University of Leicester, Leicester, LE1 6TP United Kingdom; 8 Department of Nutrition and Dietetics, School of Health Science and Education, Harokopio University of Athens, Athens 176-71, Greece; 9 Department of Mathematical Sciences, Norwegian Institute of Science and Technology, Trondheim 7491, Norway; 10 Department of Biology, The Bioinformatics Centre, University of Copenhagen, 2200 Copenhagen, Denmark; 11 Section for Metabolic Genetics, Novo Nordisk Foundation Center for Basic Metabolic Research, University of Copenhagen, Copenhagen 2200, Denmark; 12 Human Genetics Foundation, University of Torino, Torino IT-10126, Italy; 13 Department of Biomedical Informatics, Harvard Medical School, Boston 02115, MA USA; 14 Oxford Centre for Diabetes, Endocrinology and Metabolism, Radcliffe Department of Medicine, University of Oxford, Old Road, Headington, Oxford, OX3 7LE United Kingdom; 15 William Harvey Research Institute, Barts and The London School of Medicine and Dentistry, Queen Mary University of London, London, EC1M 6BQ United Kingdom; 16 Department of Haematology, Cambridge Biomedical Campus, University of Cambridge, Long Road, Cambridge, CB2 0PT United Kingdom; 17 The National Institute for Health Research Blood and Transplant Unit (NIHR BTRU) in Donor Health and Genomics at the University of Cambridge, Strangeways Research Laboratory, Wort’s Causeway, University of Cambridge, Cambridge, CB1 8RN United Kingdom; 18 MRC/BHF Cardiovascular Epidemiology Unit, Department of Public Health and Primary Care, Wort’s Causeway, University of Cambridge, Strangeways Research Laboratory, Cambridge, CB1 8RN United Kingdom; 19 British Heart Foundation Centre of Excellence, Division of Cardiovascular Medicine, Addenbrooke’s Hospital, Hills Road, Cambridge, CB2 0QQ United Kingdom; 20 Anogia Medical Centre, 740 51 Anogia, Greece

Correction to: *Nature Commununications*; 10.1038/s41467-018-07070-8, published online 7 Nov 2018

The original version of this Article contained an error in Fig. 2. In panel a, the two legend items “rare” and “common” were inadvertently swapped. This has been corrected in both the PDF and HTML versions of the Article.

