# Supplementary material for: Cohort-wide deep whole genome sequencing and the allelic architecture of complex traits
Source: Nat Commun. 2018 Nov 7;9:4674. doi: 10.1038/s41467-018-07070-8 (PMC6220258; doi:10.1038/s41467-018-07070-8)

“Cohort-wide deep whole genome sequencing and the allelic architecture of complex traits”

Gilly *et al.*

SUPPLEMENTARY INFORMATION

Supplementary Tables

Supplementary Table 1
Region definition, variant selection and weighting systems used to define testing conditions for burden analysis.

Burden analysis condition	Weighting system	Criterion	Exons	Regulatory Regions
LOFTEE HC	none	Predicted LoF by LOFTEE with high confidence	yes	no
LOFTEE LC	none	Predicted LoF by LOFTEE, both high and low confidence	yes	no
Exon severe	none	Ensembl most severe consequence more severe than missense	yes	no
Exon CADD	CADD	none	yes	no
Exon CADD median	CADD	CADD>5.851	yes	no
Exon+50 CADD	CADD	none	yes extended by 50bp	no
Exon+Regulatory Eigen	Eigen (raw score + 1)	Eigen>0	yes extended by 50bp	yes
Exon+Regulatory EigenPhred	Phred-transformed Eigen	none	yes extended by 50bp	yes
Exon+Regulatory EigenPCPhred	Phred-transformed EigenPC	none	yes extended by 50bp	yes
Regulatory only EigenPhred	Phred-transformed Eigen	none	no	yes

Supplementary Table 2

Burden test *P*-values for adiponectin levels in the *ADIPOQ* gene, conditioned on known adiponectin and diabetes-associated variants.

rsID	position (GRCh38) on chromosome 3	previous association	conditioned burden *P*-value
rs16861329	186948673	type 2 diabetes	4.76E-08
rs17366568	186852664	adiponectin levels	3.79E-08
rs182052	186842993	adiponectin levels	4.79E-08
rs822387	186838248	adiponectin levels, with and without BMI adjustment	2.56E-07
rs864265	186836503	adiponectin levels	5.56E-08
rs1648707	186833922	adiponectin levels	4.68E-08
rs10937273	186831906	adiponectin levels	5.24E-08
rs6810075	186830776	adiponectin levels	4.77E-08
rs266717	186812695	adiponectin levels	4.46E-08
rs266719	186783859	adiponectin levels	2.40E-07
rs822354	186762417	adiponectin levels	6.57E-08
rs74577862	186843903	adiponectin levels	1.2E-07
rs201813484	186841095	adiponectin levels	3.1E-07

Supplementary Table 3

Fraction of SNVs with |iHS|>2 in *APOC3*, *UGT1A9*, *ADIPOQ* and *FAM189B* compared to all other genes. For each gene its fraction of SNVs with |iHS|>2 is given in parenthesis and the percentile in the empirical distribution of these fractions for all genes using four different definitions of the genomic region representing the genes. We mainly considered the most inclusive definition (the bottommost), but included the others for comparison to assess robustness to this definition. For *FAM189B* the percentile is also given for the subset of genes with a similar gene length, defined as the number of SNVs with iHS values (rightmost column). A percentile of 80% means that 80% of values are less than or equal to the value.

**Definition of burden testing condition**	***APOC3* compared to all genes**	***UGT1A9* compared to all genes**	***ADIPOQ* compared to all genes**	***FAM189B* compared to all genes**	***FAM189B* compared only to genes with within +/- 10% of # SNVs in *FAM189B***
Exons only	41.0th percentile (0.00)	41.0th percentile (0.00)	41.0th percentile (0.00)	98.3th percentile (0.67)	97.4th percentile (0.67)
Exons extended by 50bp and regulatory elements	28.1th percentile (0.00)	28.1th percentile (0.00)	28.1th percentile (0.00)	97.4th percentile (0.42)	95.7th percentile (0.42)
Region spanning all exons	15.3th percentile (0.00)	15.3th percentile (0.00)	15.3th percentile (0.00)	96.7th percentile (0.32)	94.6th percentile (0.32)
Region spanning all exons extended by 50bp and regulatory elements	27.5th percentile (0.00)	27.5th percentile (0.00)	27.5th percentile (0.00)	95.6th percentile (0.33)	93.9th percentile (0.33)

Supplementary Table 4

Weighted mean F_{ST} in *FAM189B* compared to all other genes. The weighted mean F_{ST} for SNVs within *FAM189B* is given in parenthesis and the percentile of this value in the empirical distribution for all genes using four different definitions of the genomic region representing the genes (left column). In the right column, *FAM189B* is only compared to the subset of genes with a similar gene length, defined as the number of SNVs with F_{ST} values (rightmost column) within 10%.

**Definition of gene**	***FAM189B* compared to all genes**	***FAM189B* compared only to genes with within +/- 10% of # SNVs in *FAM189B***
Exons only	96.3th percentile (0.036)	96.8th percentile (0.036)
Exons extended by 50 bp and regulatory elements	99.1th percentile (0.050)	99.7th percentile (0.050)
Region spanning all exons	98.0th percentile (0.042)	97.5th percentile (0.042)
Region spanning all exons extended by 50 bp and regulatory elements	99.0th percentile (0.042)	100th percentile (0.045)

Supplementary Table 5
Number of genes with at least 2 SNVs for the different burden analysis conditions.

**Analysis condition**	**Number of genes**
GENCODE V25 (all protein-coding, not tested)	18,997
LOFTEE HC	85
LOFTEE LC	1,727
Exon severe	7,660
Exon CADD	18,428
Exon CADD median	18,138
Exon+50 CADD	18,551
Exon+Regulatory Eigen	18,961
Exon+Regulatory EigenPhred	18,660
Exon+Regulatory EigenPCPhred	18,722
Regulatory only EigenPhred	17,607

Supplementary Table 6
Broad functional categories are defined by grouping together several variant categories as defined by Ensembl VEP.

Attributed category	Ensembl VEP functional class
LoF (high)	*(See Supplementary Figure 4)*
LoF (low)	*(See Supplementary Figure 4)*
severe	start_lost<br>stop_gained<br>stop_lost<br>frameshift_variant<br>transcript_ablation
intergenic	intergenic_variant
Intronic	intron_variant
UTR	3_prime_UTR_variant<br>5_prime_UTR_variant
Up/Down stream	upstream_gene_variant<br>downstream_gene_variant
splice variant	splice_donor_variant<br>splice_acceptor_variant<br>splice_region_variant
synonymous	synonymous_variant
other coding	coding_sequence_variant<br>incomplete_terminal_codon_variant<br>initiator_codon_variant<br>missense_variant<br>stop_retained_variant<br>inframe_deletion<br>inframe_insertion
other noncoding	nc_transcript_variant<br>non_coding_transcript_exon_variant<br>non_coding_exon_variant<br>mature_miRNA_variant<br>non_coding_transcript _variant
regulatory	regulatory_region_variant<br>TF_binding_site_variant<br>TFBS_ablation

Supplementary Figures

Supplementary Figure 1

Sequencing depth distribution of 1,457 MANOLIS samples. The box indicates quartiles and the centre line is the median. Whiskers extend to 1.5x the interquartile range. The mean is 22.5x and the median is 21.9x. Sequencing depths range from 14.7x to 40x.

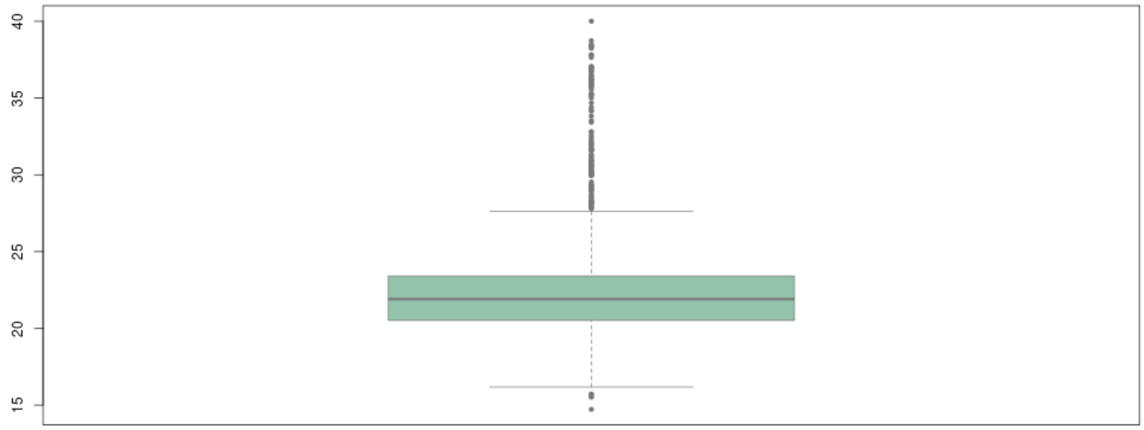

Supplementary Figure 2

True positive rate for NA12878 at various sequencing depths. Compared to Genome in a Bottle 0.2 for SNVs and INDELs. Genome-wide, a single downsampling replicate was performed for each depth.

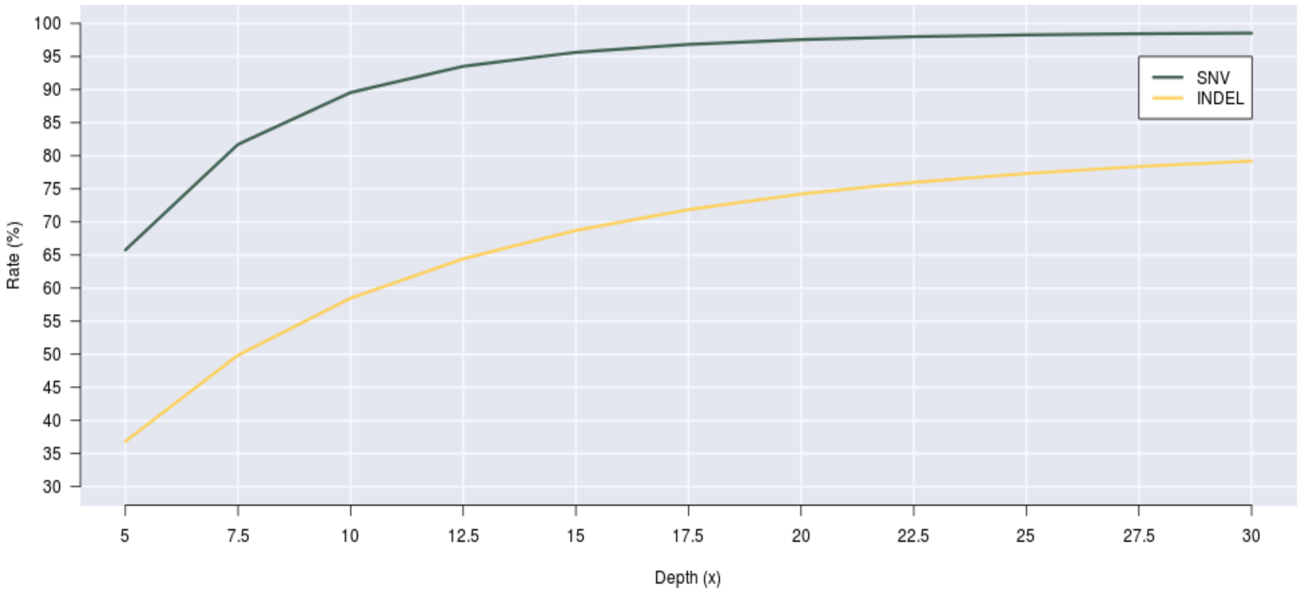

Supplementary Figure 3
Unique and shared SNVs between 30x and 22.5x depth whole genome sequencing.

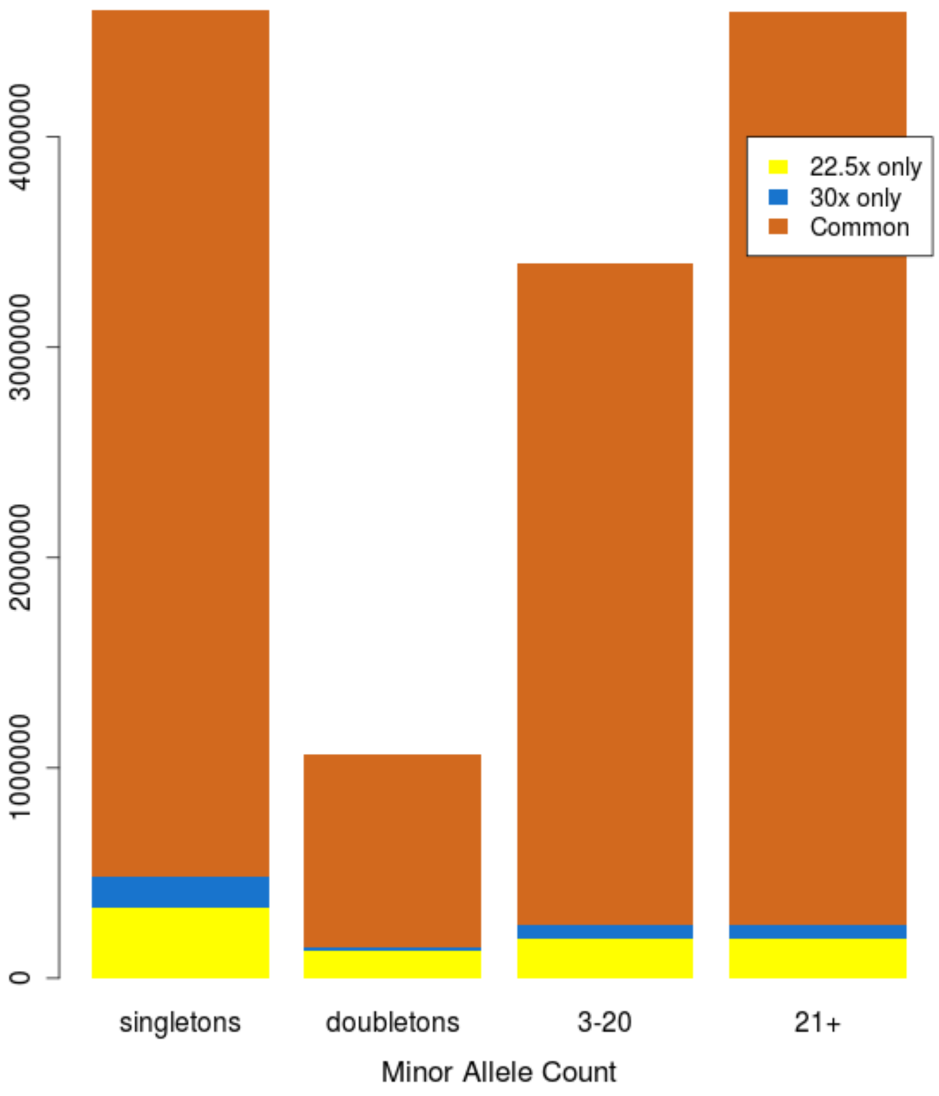

Supplementary Figure 4

Ensembl most severe consequence for loss-of-function variants. Breakdown is shown for variants predicted as loss-of-function with (a) high-confidence (HC) and (b) low-confidence (LC) by LOFTEE, along with genome-wide counts per Ensembl predicted consequence.

a.

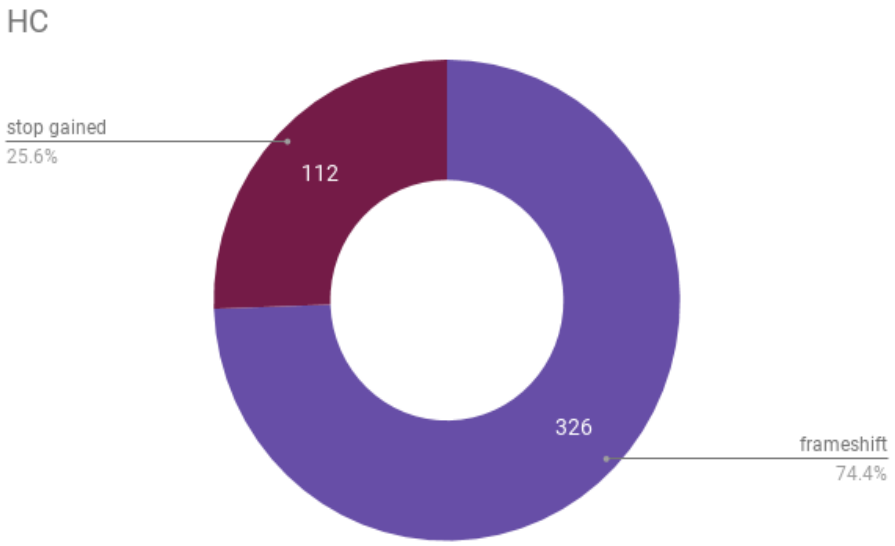

b.

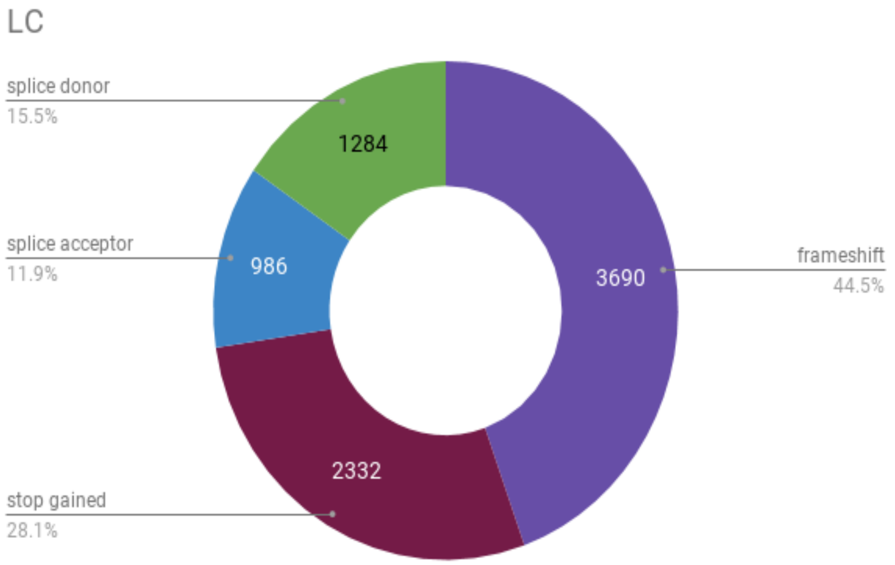

Supplementary Figure 5

Distributions of rare variant counts compared to cosmopolitan populations. (a) singleton and (b) doubleton counts in 1,000 draws of 100 MANOLIS samples (blue histograms). The vertical orange line indicates the observed count in 100 TEENAGE samples downsampled to 22.5x. Red lines are fitted normal distributions. (c) rare variant counts in MANOLIS (blue line) and INTERVAL (boxplots) in 500 draws of 1,482 samples from INTERVAL. Boxes extend from the 1st to the 3rd quartiles, whiskers extend to 1.5 times the interquartile range. Centre lines are the median.

a.

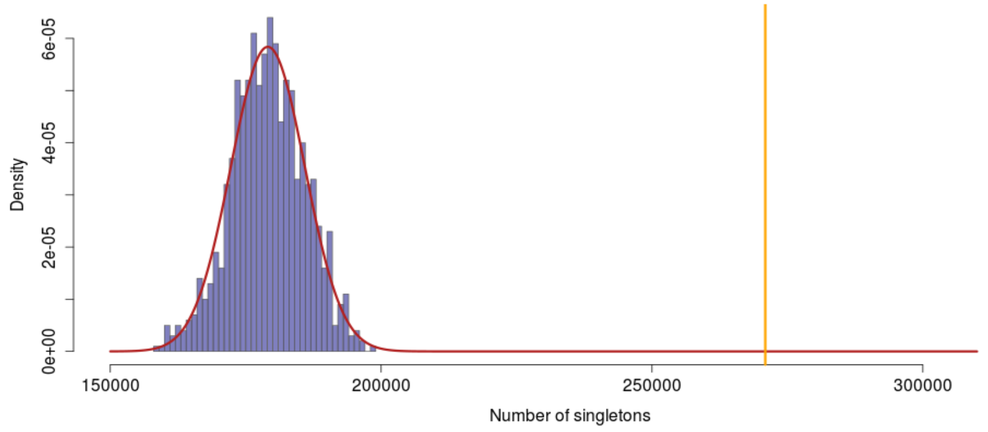

b.

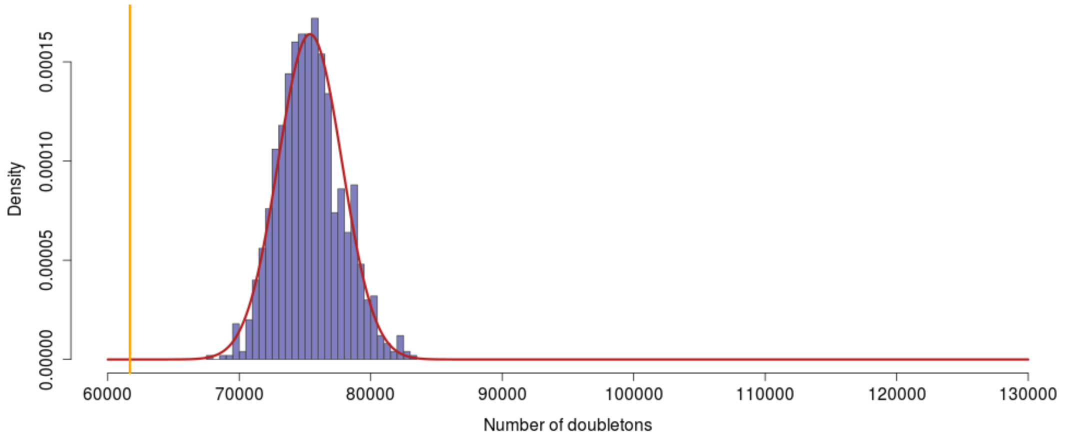

c.

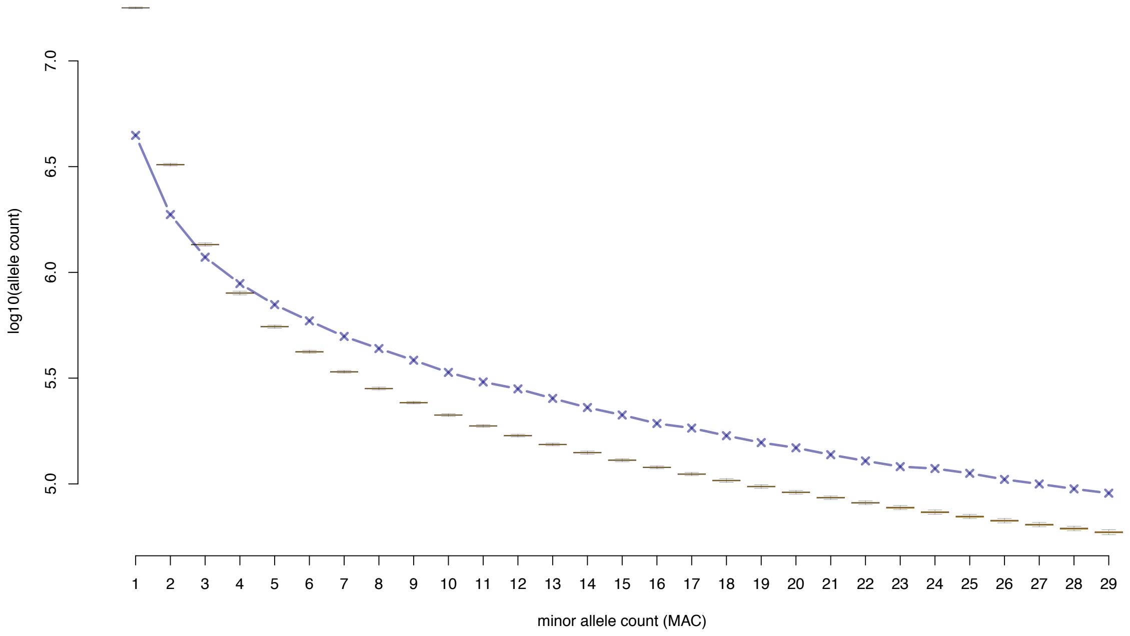

Supplementary Figure 6

Frequencies and functional annotations of all novel variants in MANOLIS. (a) depicts frequency bins and (b) variant consequences. Novelty is established by comparing variant location and alleles to Ensembl VEP annotation as well as gnomAD genomic variants lifted-over to build 38.

a.

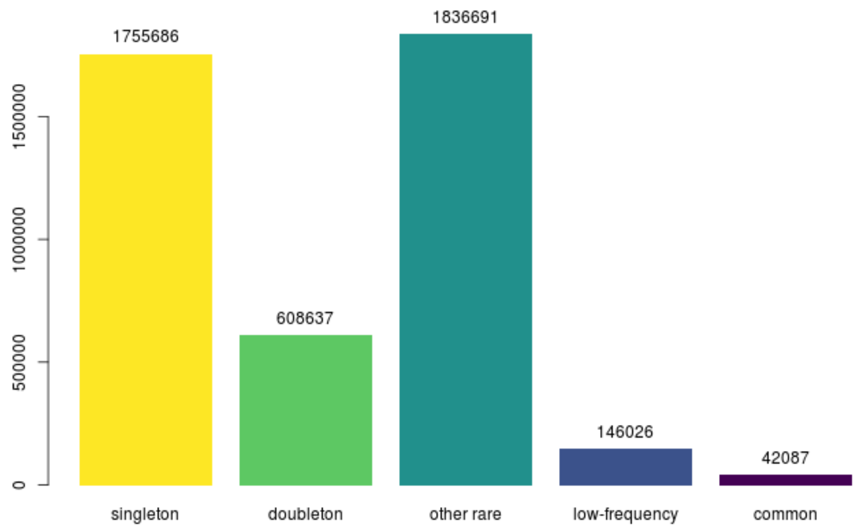

b.

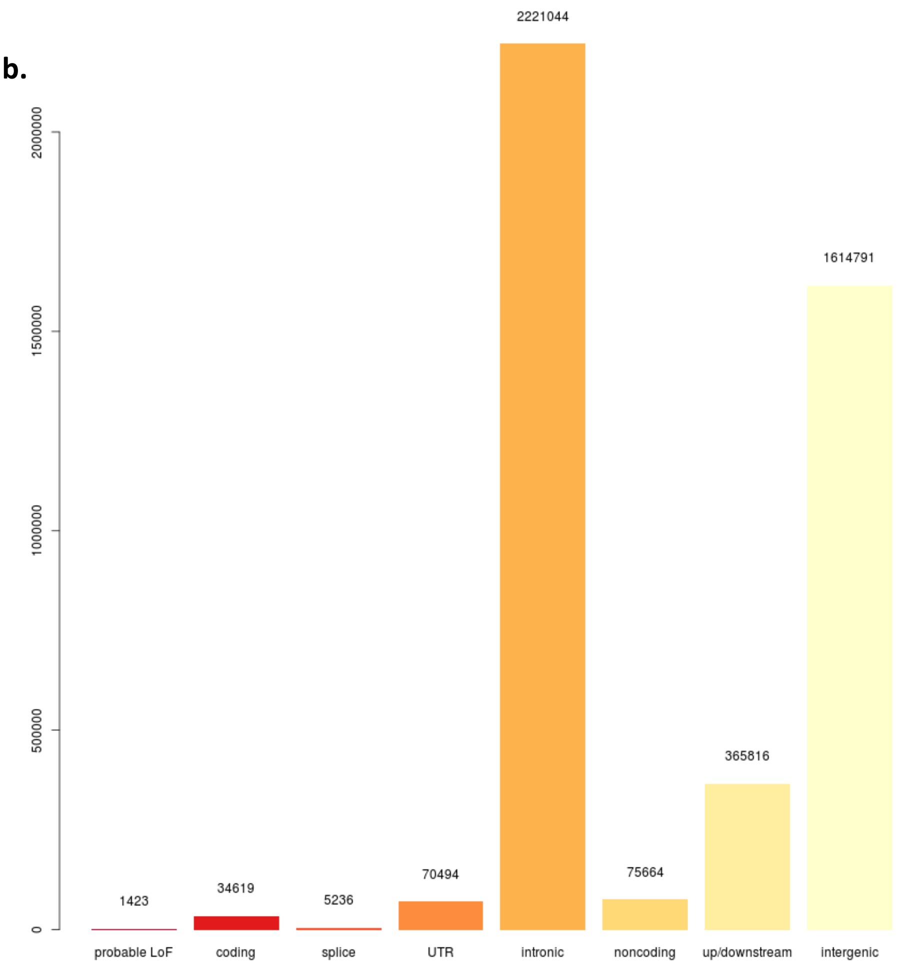

Supplementary Figure 7

Correlogram of z-scores arising from all evaluated burden testing scenarios. Quantities reported are Pearson's correlations of z-transformed *P*-values across all gene-trait pairs for all six tested traits. For each cell, *P*-values of all gene-trait pairs that were tested in both conditions are included. Clusters were generated using hierarchical clustering. The exon LoF HC scenario is not included in the correlogram due to the low number of genes containing more than one high-confidence loss-of-function variant (n=85).

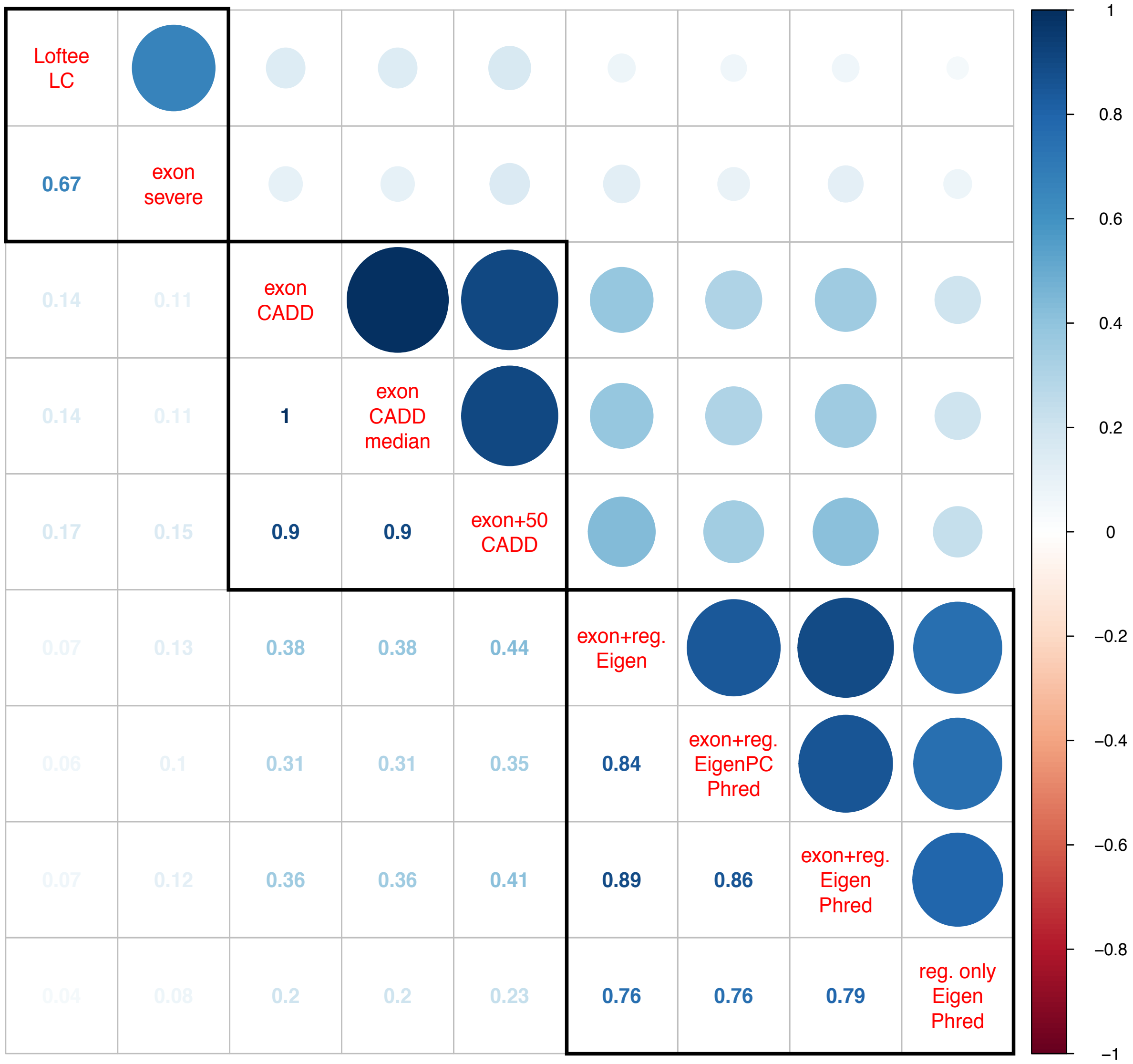

Supplementary Figure 8

Evidence for association for the 20 study-wide significant trait-gene pairs across all tested conditions. *P*-values are on the -$\log_{10}$ scale. Grey cells indicate that an insufficient number of variants passed the inclusion threshold. Dark orange denotes study-wide significance and turquoise green denotes suggestive association.

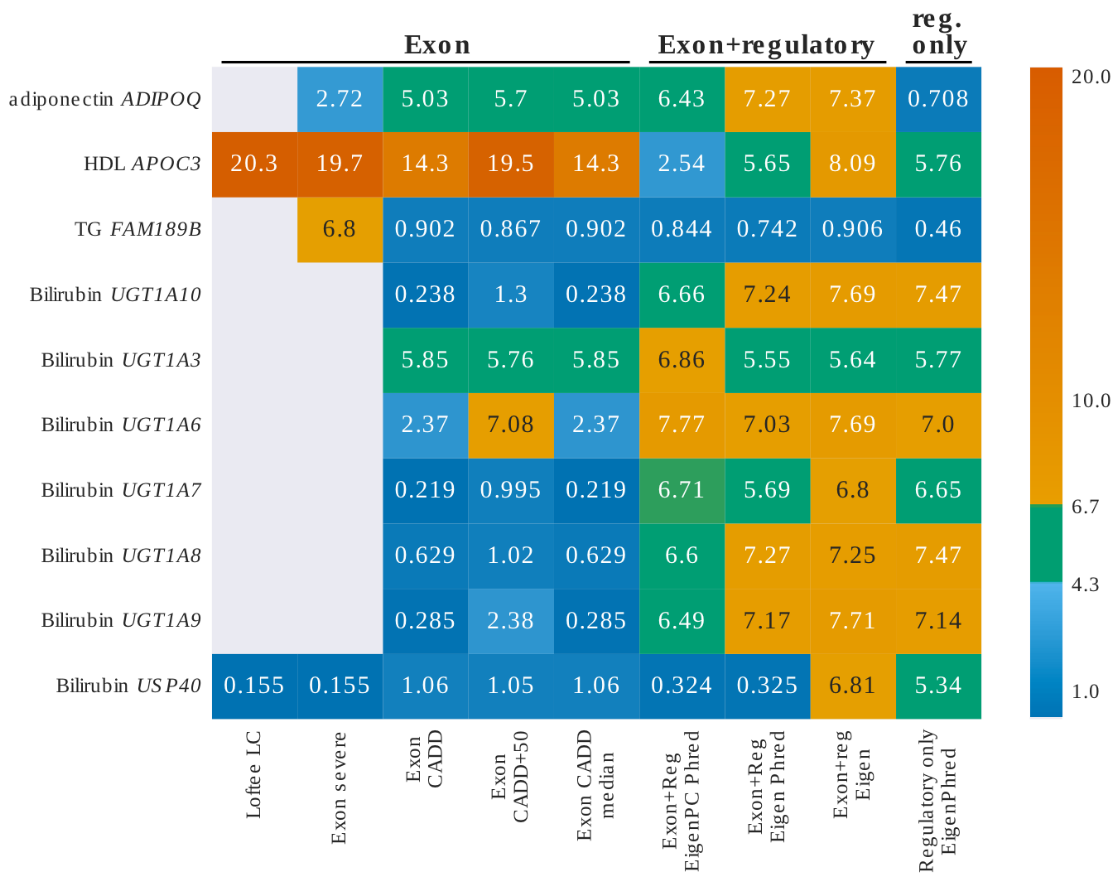

Supplementary Figure 9

Comparison of kinship coefficients produced by different methods. KING and EMMAX estimates on the y axis are compared to those produced by GEMMA[24] on the x axis. All kinship coefficients are calculated using the same dataset (MAF>5%, missingness<1%, LD-pruned). IBS coefficients (KING Related, EMMAX IBS and Plink) are both higher on average and less sensitive to increased relatedness than their Balding-Nichols counterparts (GEMMA, EMMAX BN and KING homogenous). Red lines represent OLS regression slopes.

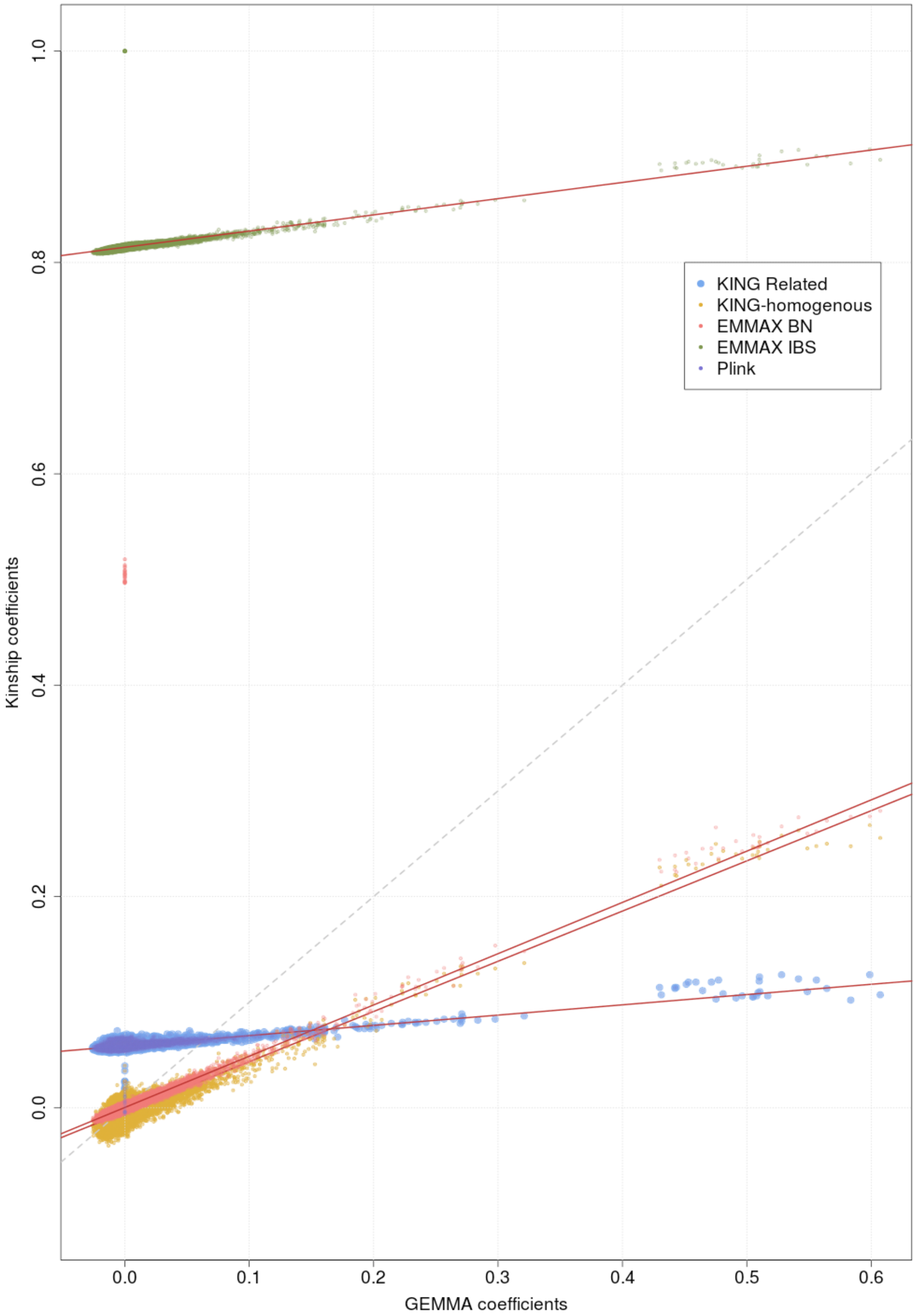

Supplementary Figure 10

QQ-plots for all tested conditions across all analysed traits. The lambda values are displayed next to the condition name in the legend. lambda is calculated as

$$\lambda_{\mathrm{GC}} = \frac{\mathrm{median}(-log_{10}(p))}{-log_{10}(0.5)}$$

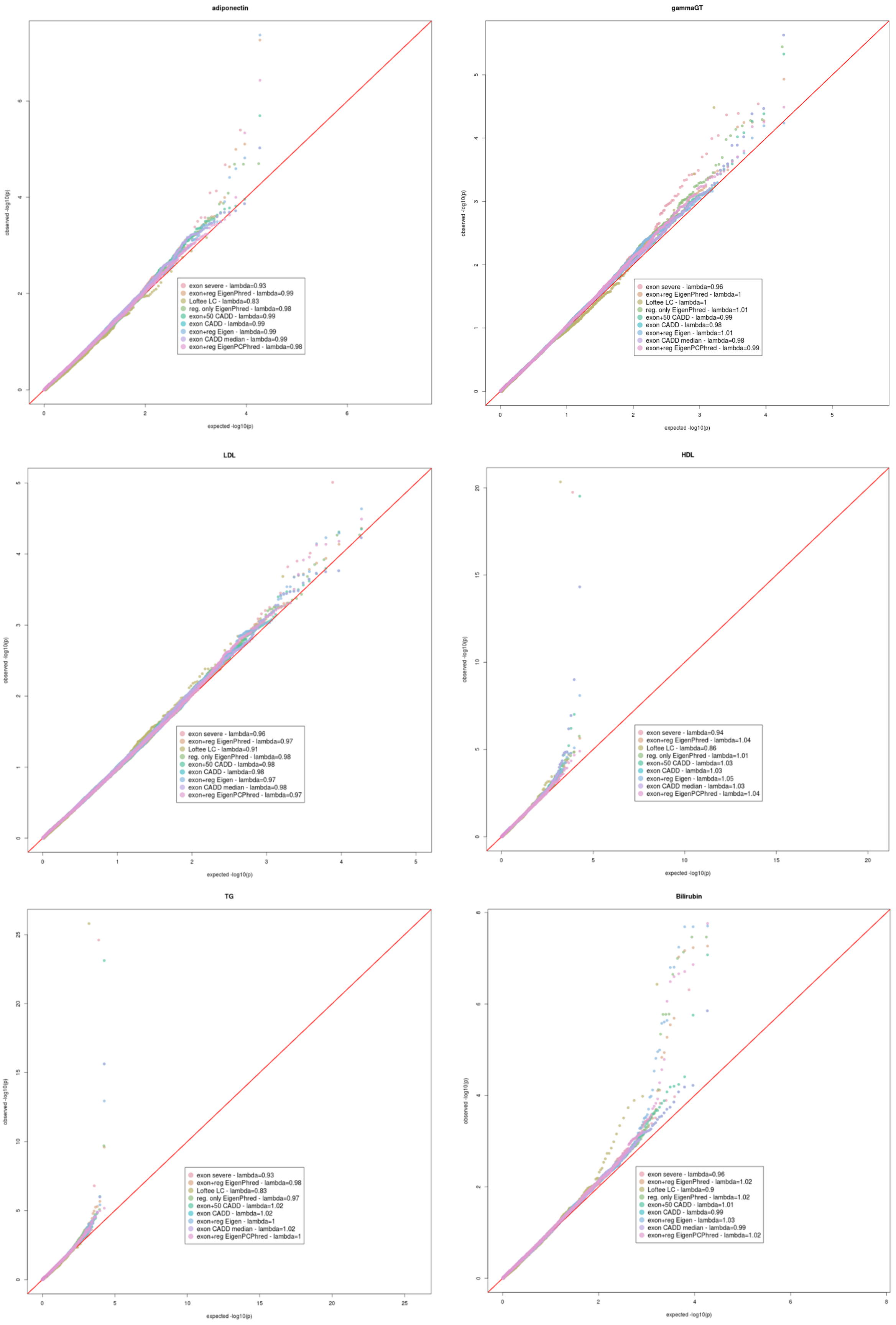

Supplement: Supplementary file 3 — Supplementary Information [file 41467_2018_7070_MOESM3_ESM.pdf]
