# Supplementary material for: Cohort-wide deep whole genome sequencing and the allelic architecture of complex traits
Source: Nat Commun. 2018 Nov 7;9:4674. doi: 10.1038/s41467-018-07070-8 (PMC6220258; doi:10.1038/s41467-018-07070-8)
Supplement: Supplementary file 2 — Description of Additional Supplementary Files [file 41467_2018_7070_MOESM2_ESM.pdf]

Reviewer #1 (Remarks to the Author):

Gilly et al report the results of performing a burden association study on a relatively small set of individuals from an isolated population. Their phenotypic test set is limited to only six cardiometaembolic traits. Their key result is a marginally signficiant burden association between variants in FAM189A and trigliceride levels. Secondarily, the authors provide some evidence for rare variants associating at four loci previously associated through common variant association.

The authors sometimes refer to FAM189A and somtimes FAM189B. I believe they are referring to FAM189B.

The initial observation of P=1.56e-7 for FAM189B and the replication P=9.3e-3 are very marginal. Was the same burden test employed in both instances?

Did the authors do any genomic control adjustment? E.g. LD score regression or adjustment based on median chi-squared values. The baseline relatedness in an isolate like this might enduce a non-trivial correction factor which could affect the significance of some of the borderline associations.

As the authors state in the results, the UGT1A9 burden association is probably a shadow of the association of common variants at the locus. This should be made clear in the presentation of results in the abstract or, alternatively, the UGT1A9 result should be removed from the abstract.

The ADIPOQ association is again quite marginally signficant. Again, the evidence for an independant association with rare variants.

The GGT1 association is a cis-association with gammaglutamyltransferase (GGT) levels and therefore not un-expected. Indeed, as the authors point out, common variants at the locus have already been associated with GGT levels. However, the association of the authors in not signficant and provides very marginal evidence for additional markers associating with GGT at the locus. GGT1 should be removed from the abstract.

Have the authors attempted taking the loci of all common variants associating with their six traits to see if any of these show evidence of secondary rare signals?

The conclusion of the authors that „it is possible to achieve near-perfect sensitivity and quality for rare variant calling and genotyping with half the depth“ is hard to understand given that the quality of indel calls is substantially lower for indels (Figure 1) even at 22.5x, especially given that indels represent over half of all loss-of-function variants.

Minor points:

Why only use 2 million randomly selected variants in table 2, rather than all the available variants? Are these the high confidence or low confidence variants?

Define \sigma on first use.

The number of signficant digits provided in P-values varies.

When specifying effects what does „\beta = 2.59 \sigma = 0.57“ mean? I.e. what are the units? What is \sigma?

Reviewer #2 (Remarks to the Author):

In this article, Gilly and colleagues performed high-coverage whole-genome sequencing (WGS) on 1457 individuals from an isolated Greek population to explore the role of rare genetic variants on 6 complex human traits. Although the authors argue that there is still a debate on the role that this class of genetic variation plays in the architecture of complex human phenotypes, I believe that the community generally agrees on its importance. Indeed, there has been a number of high-profile publications on this exact same topic in the last 2-3 years. It is true that the focus of the previous studies has been mostly on rare coding variation, although rare regulatory variants have also been described (e.g. PMID 26367794). Nevertheless, the manuscript by Gilly et al. is interesting because it explores different strategies to test association between rare variants and quantitative traits, and also because it reports a novel, and replicated, association with triglyceride levels. I have the following comments:

1. Isolated populations are characterized by less genetic variation, and a shift towards more frequent genetic variants. Is this what you see in MANOLIS? I understand from the text that there are more LoF in MANOLIS participants vs deCODE participants, presumably because of a more severe bottleneck in Iceland. In Supplementary Figure 5, it looks like there are more singleton but less doubletons in MANOLIS vs TEENAGE. Doubletons are still pretty rare so that is surprising to me.

2. Figure 2. It looks like the fraction of rare variants is pretty much the same for all classes of annotations. I was expecting to see a larger fraction of rare variants among coding variants because of negative selection. How do you define coding variants? Do you include synonymous variants? Could you add a bar in this plot for predicted LoF – certainly there we would expect to see a larger fraction of rare variants.

3. Figure 2 and Supplementary Figure 6 show that there are more intronic than intergenic variants. Is this expected given that a larger fraction of the human genome is intergenic?

4. For my points 1-3 above, it would be interesting to compare with the INTERVAL WGS data.

5. I read the legend of Figure 3 several times, and I am still very confused. I don't understand why the horizontal lines have different length, and what are the large grey dots (variants not tested)? Are the grey dots previous GWAS sentinel variants for these traits?

6. Rare variant tests have a tendency to not be well-calibrated, especially for very rare variants. That is something that we see even for SKAT-O when the sample size is modest (as here). I would like to see QQ plots for the different analyses performed to get a sense of inflation/deflation of the test statistics.

7. The very (too) short paragraph on selection would gain interest by providing a brief introduction on what iHS actually measures and what does it reflect in terms of selection. Why using a cutoff of iHS>2? Is the iHS result consistent with other selection metrics (e.g. Fst)? And what is known about selection at this FAM189B locus across diverse populations (e.g. 1000G data)?

8. I find the discussion on selection rather weak and the article might benefit from a longer discussion of the population genetics of this cohort (although this might have been done elsewhere; if that is the case, please summarize in the introduction).

Response to Referees

Reviewer #1:

Major:

1. The authors sometimes refer to *FAM189A* and sometimes *FAM189B*. I believe they are referring to *FAM189B*.

Thank you for pointing out this typographical error, the gene is indeed *FAM189B.*

2. The initial observation of P=1.56e-7 for *FAM189B* and the replication P=9.3e-3 are very marginal. Was the same burden test employed in both instances?

This is an important point, which we have now further highlighted in the manuscript. We expected allelic heterogeneity with respect to the rare variant composition of burden associations, partly due to the different population genetics characteristics of the studied populations and partly due to the rare frequency of the contributing variants. This has been well-documented by theoretical and empirical studies. For example, different rare variants contribute to a burden signal in the *APOC3* gene associated with blood triglyceride levels in different populations[1-3]. We used the same array of tests for discovery and replication, to ensure that replication was agnostic in terms of burden category. For example, our replication strategy would have allowed us to detect a regulatory-only burden in the replication cohort, even if the discovery signal had been exonic in nature. However, this pattern was never observed, and replication was always consistently within the same broad functional class as discovery. This is an important point to make, and we have added it to the paragraph discussing replication (added text in italics):

"We replicate the *FAM189B* association in an independent dataset with deep whole genome sequence data, in which the disruptive rare alleles are also associated with the same trait in the same direction. Across the board, we replicate all burden signals for which replication cohort trait measurements are available. We find that allelic heterogeneity is prevalent, partly due to the rare nature of the variants contributing to the burdens, and partly due to the distinct population genetics characteristics of the discovery and replication sets. *All of the burdens reported here have strongest evidence for replication in slightly different testing conditions from the discovery, although consistently within the same broad functional class.* These findings have important consequences for defining replication in sequence-based studies of rare variants, and highlight the importance of defining replication at the locus level rather than the variant level for burden signals."

3. Did the authors do any genomic control adjustment? E.g. LD score regression or adjustment based on median chi-squared values. The baseline relatedness in an isolate like this might induce a non-trivial correction factor which could affect the significance of some of the borderline associations.

Accounting for relatedness is indeed a critical requirement when analysing genomic data in isolated populations. The method we used to test for rare variant burden signals (called MONSTER) includes an empirical genetic relatedness matrix in its model, in order to take relatedness into account. We discuss relatedness matrices using different metrics in the Methods and Supplementary Figure 9.

4. As the authors state in the results, the UGT1A9 burden association is probably a shadow of the association of common variants at the locus. This should be made clear in the presentation of results in the abstract or, alternatively, the UGT1A9 result should be removed from the abstract.

We have removed the *UGT1A9* from the abstract:

"We find evidence of rare variant burdens *that are independent of established common variant signals* (*ADIPOQ* and adiponectin, P=4.2x10^{-8}; *APOC3* and triglyceride levels, P=1.5×10^{-26}), *and identify* replicating evidence for a burden associated with triglyceride levels in *FAM189B* (P=2.2x10^{-8}), indicating a role for this gene in lipid metabolism."

5. The ADIPOQ association is again quite marginally significant. Again, the evidence for an independent association with rare variants.

The *ADIPOQ* signal, similarly to the *GGT1* association, is a *cis* association and approximately one order of magnitude stronger than the corrected significance threshold. In this population, no common variant signal is present at this locus. Previously associated SNVs are either not present in the MANOLIS cohort or not associated with adiponectin levels. Nevertheless, we have conditioned the observed burden association on the genotypes of all variants with previously reported associations for adiponectin, type 2 diabetes or obesity that are polymorphic in MANOLIS (Supplementary Table 4) and show conditional independence of our burden signal.

6. The GGT1 association is a cis-association with gamma glutamyltransferase (GGT) levels and therefore not un-expected. Indeed, as the authors point out, common variants at the locus have already been associated with GGT levels. However, the association of the authors in not significant and provides very marginal evidence for additional markers associating with GGT at the locus. GGT1 should be removed from the abstract.

We agree with the reviewer that the association is not unexpected, and believe that this provides a nice proof of principle. As per the reviewer's suggestion, we have now removed the *GGT1* burden from the abstract.

7. Have the authors attempted taking the loci of all common variants associating with their six traits to see if any of these show evidence of secondary rare signals?

We examined all rare variant burden associations with suggestive significance ($p<5x10^{-5}$) for all six traits, and do not find evidence of rare variant signals at established loci beyond the burdens that are described in the article.
We have made this clear in the manuscript:
"*We examined rare variant burden associations with suggestive significance ($P<5x10^{-5}$) across the six traits under investigation, and do not find evidence of further rare variant signals at established loci.*"

8. The conclusion of the authors that „it is possible to achieve near-perfect sensitivity and quality for rare variant calling and genotyping with half the depth "is hard to understand given that the quality of indel calls is substantially lower for indels (Figure 1) even at 22.5x, especially given that indels represent over half of all loss-of-function variants.

We agree with the reviewer and have amended the text to clarify that our statement refers to SNVs:
"*It is possible to achieve near-perfect sensitivity and quality for rare SNV calling and genotyping with half the depth.*"
We have further highlighted that INDEL quality and call rate are less robust to depth decreases between 30x and 15x:
"*This observation does not extend to INDELs, for which depth increases above 15x can result in a 15% increase in genotype quality and a 40% increase in true positive rate.*"

Minor:

9. Why only use 2 million randomly selected variants in table 2, rather than all the available variants? Are these the high confidence or low confidence variants?

The analysis had been run on a randomly selected, genome-wide representative subset of variants for computational efficiency. We have now repeated the analysis using all variants genome-wide. We have updated Figure 2 and Supplementary Table 7 to delineate variant consequences more finely. In particular, we now separate high- and low-confidence LoF variants from other coding consequences.

10. Define \sigma on first use.

We have defined σ at first mention.

11. The number of significant digits provided in P-values varies.
We have harmonised p-values throughout the article.

12. When specifying effects what does „\beta = 2.59 \sigma = 0.57" mean? I.e. what are the units? What is \sigma?
We have defined σ and β at first mention and expressed the units of β throughout the manuscript (units of standard deviation).

Reviewer #2:

General comment:
Although the authors argue that there is still a debate on the role that this class of genetic variation plays in the architecture of complex human phenotypes, I believe that the community generally agrees on its importance. Indeed, there has been a number of high-profile publications on this exact same topic in the last 2-3 years. It is true that the focus of the previous studies has been mostly on rare coding variation, although rare regulatory variants have also been described (e.g. PMID 26367794). Nevertheless, the manuscript by Gilly et al. is interesting because it explores different strategies to test association between rare variants and quantitative traits, and also because it reports a novel, and replicated, association with triglyceride levels.
We thank the reviewer for their comment.

Major and Minor:
1. Isolated populations are characterized by less genetic variation, and a shift towards more frequent genetic variants. Is this what you see in MANOLIS? I understand from the text that there are more LoF in MANOLIS participants vs deCODE participants, presumably because of a more severe bottleneck in Iceland. In Supplementary Figure 5, it looks like there are more singleton but less doubletons in MANOLIS vs TEENAGE. Doubletons are still pretty rare so that is surprising to me.

Due to the sample size difference between MANOLIS and TEENAGE (an order of magnitude), we are unable to directly compare singleton and doubleton counts between the two cohorts. To address this, we extracted random samples of 100 individuals from the MANOLIS cohort to match the sample size of TEENAGE. The enrichment of singletons and depletion of doubletons is therefore to be interpreted with respect to this reduced sample size (they correspond to MAF<0.005 and 0.005<MAF<0.01, respectively). We have substantially expanded the section describing subsampling methods and implications for minor allele frequencies:
"***Rare variant counts in MANOLIS, TEENAGE and INTERVAL***
Since sample sizes differ between the three datasets, we randomly subsampled the larger dataset to a matching size for each pairwise comparison. We used these resampled datasets to build empirical distributions for rare variant counts in the larger dataset, and compared it to counts in the smaller dataset. TEENAGE (n=100) was smaller compared to MANOLIS (n=1,482), so we drew 1,000 sets of 100 samples from the MANOLIS study for the comparison. We counted 270,916 singletons and 61,690 doubletons in TEENAGE, compared with a median of 179,100 (p=1.4x10^{-94}) and 75,280 (P=3.0x10^{-19}), respectively, in MANOLIS (Supplementary Figure 5). For n=100, singletons correspond to MAF<0.005 and doubletons to 0.005<MAF<0.1.
For the INTERVAL (n=3,742) comparison, MANOLIS was the smaller dataset, so we resampled 500 sets of 1,482 samples from the INTERVAL cohort and counted variants up to MAC=29 (MAF=0.01). The increased resolution provided by this larger sample size shows that rare variant counts are

greater in the cosmopolitan population below MAC=4 (MAF=0.0013), but greater in the isolate for 0.0013<MAF<0.1, consistent with our coarser observation in TEENAGE (Supplementary Figure 5.c). For p-values of singleton counts, empirical quantiles cannot be computed for such large deviations from the mean. We fitted a normal distribution to singleton counts, and computed the theoretical quantile corresponding to the observed count in the smaller cohort."

2. Figure 2. It looks like the fraction of rare variants is pretty much the same for all classes of annotations. I was expecting to see a larger fraction of rare variants among coding variants because of negative selection. How do you define coding variants? Do you include synonymous variants? Could you add a bar in this plot for predicted LoF – certainly there we would expect to see a larger fraction of rare variants.

Thank you for this suggestion. We have now broken down functional variant consequences into finer categories, outlining both classes of predicted loss-of-function variants, splice variants and severe-consequence variants among coding SNVs. As expected by the reviewer, an enrichment of rare variants is detectable for both classes of predicted LoF variants, as well as to a lower extent in the severe and "other coding" categories. This latter class of variants contains SNVs with Ensembl most severe consequence among the following: "coding_sequence_variant", "incomplete_terminal_codon_variant", "initiator_codon_variant", "missense_variant", inframe_deletion", "inframe_insertion" and "protein_altering_variant". We have updated Table 8 to show the full breakdown of functional classes displayed in this plot.

3. Figure 2 and Supplementary Figure 6 show that there are more intronic than intergenic variants. Is this expected given that a larger fraction of the human genome is intergenic?

This is a good point. We used genome-wide annotations of Ensembl most severe consequences. Ensembl is liberal in its functional annotation, with "intergenic" being the least severe consequence in the hierarchy. Therefore, whenever possible, variants are attributed a non-intergenic consequence. Gene annotations are not restricted to protein-coding genes, but also include RNA genes and pseudogenes. Using Ensembl's definition for genic regions, we find that 56% of the genome overlaps with elements annotated as genes. Adding to that up- and downstream regions and regulatory regions, we obtain a number of intergenic variants that is likely to be underestimated. We have updated the legend of Figure 2 to reflect this bias in Ensembl's most severe consequence calculation:
"*The number of intergenic variants is likely to be an underestimate based on Ensembl's most severe consequence annotation.*"

4. For my points 1-3 above, it would be interesting to compare with the INTERVAL WGS data.

Thank you for this suggestion. We have now performed this additional analysis and have updated Figure 2 to reflect the frequency per consequence makeup of all variants in INTERVAL as well. We observe a similarly increased proportion of rare variants among severe categories. This observation is now also made in the main text:
"We observe an enrichment of rare variants among the coding and splice variant categories in MANOLIS (P=9.5x10^{-16}), and we recapitulate this in an independent dataset of 3,724 individuals with whole genome sequencing from the UK-based INTERVAL cohort[4](Figure 2)."
We have also computed 500 resamplings of 1,482 samples from the INTERVAL cohort in order to compare rare variant counts between MANOLIS and INTERVAL. We now report the singleton depletion p-value in the main text ("*We also observe a lower rate of singletons compared to the general Greek population and the INTERVAL cohort ($P \approx 10^{-167}$ and $P < 10^{-200}$ respectively, one-sided empirical P-value)*"), and more detailed results are described in the updated Supplementary Figure 5, and in an independent section of the methods:
"Rare variant counts in MANOLIS, TEENAGE and INTERVAL

Since sample sizes differ between the three datasets, we randomly subsampled the larger dataset to a matching size a number of times for each pairwise comparison. We used these resampled datasets to build empirical distributions for rare variant counts in the larger dataset, and compared it to counts in the smaller dataset. TEENAGE (n=100) was smaller compared to MANOLIS (n=1,482), so we drew 1,000 sets of 100 samples from the MANOLIS study for the comparison. We counted 270,916 singletons and 61,690 doubletons in TEENAGE, compared with a median of 179,100 (p=1.4x10^{-94}) and 75,280 (P=3.0x10^{-19}), respectively, in MANOLIS (Supplementary Figure 5a, b.). For n=100, singletons correspond to MAF<0.005 and doubletons to 0.005<MAF<0.1.
For the INTERVAL (n=3,742) comparison, MANOLIS was the smaller dataset, so we resampled 500 sets of 1,482 samples from the INTERVAL cohort and counted variants up to MAC=29 (MAF=0.01). The increased resolution provided by this larger sample size shows that rare variant counts are greater in the cosmopolitan population below MAC=4 (MAF=0.0013), but greater in the isolate for 0.0013<MAF<0.1, consistent with our coarser observation in TEENAGE (Supplementary Figure 5.c).”

5. I read the legend of Figure 3 several times, and I am still very confused. I don’t understand why the horizontal lines have different length, and what are the large grey dots (variants not tested)? Are the grey dots previous GWAS sentinel variants for these traits?

We apologise for the confusing legend. We have changed it to better explain graphical elements:
"Red lines denote the burden P-value and extend over the genomic region (gene, or gene and regulatory regions) in which variants are selected for inclusion in the test. Purple lines indicate the conditioned P-value for the variant described in the text (variants rs887829, rs62625753 and rs3859862 in c., d., e. respectively). Small grey dots indicate single-point P-value for variants in the region not included in the test. Larger coloured dots represent variants included in the test, with size and colour proportional to the score used in the most significant test (Supplementary Tables 1 and 2). When no weights are applied (a. and b.), included variants are coloured blue-green. In the “Selected genomic features” panel, green bars below the gene denote regulatory regions associated with the gene."

6. Rare variant tests have a tendency to not be well-calibrated, especially for very rare variants. That is something that we see even for SKAT-O when the sample size is modest (as here). I would like to see QQ plots for the different analyses performed to get a sense of inflation/deflation of the test statistics.

We have added the QQ-plots for all analyses across all traits in a new supplementary figure (Supplementary Figure 10). Overall, the tests are well-calibrated, exhibiting some loss of power in the high-consequence runs only, in which the number of genes tested is very limited.

7. The very (too) short paragraph on selection would gain interest by providing a brief introduction on what iHS actually measures and what does it reflect in terms of selection. Why using a cutoff of iHS>2? Is the iHS result consistent with other selection metrics (e.g. F_{st})? And what is known about selection at this *FAM189B* locus across diverse populations (e.g. 1000G data)?

We agree that including more background information about iHS in the manuscript would be useful to many readers, and have added the following description of what the iHS statistic captures and why to the methods section:
“Briefly, the iHS value of an SNV becomes elevated when one of the alleles of that SNV reside on haplotypes that are longer than expected under neutrality, given the frequency of the allele. This is considered a signature of positive selection because positive selection will cause haplotypes carrying an advantageous allele to increase in frequency faster than if the allele had been neutral, which leaves less time for recombination to shorten them.”
Additionally, we have included the following sentence to the main text:
“Previous studies have shown that an elevated fraction of SNVs with |iHS|>2 in a genomic region is a signature of recent or ongoing selection”.

Furthermore, we have added the following explanation for using an a iHS threshold of 2, and supporting citations to the methods section:
"We focused on the fraction of SNVs with |iHS|>2, because previous studies have compared different methods of summarizing iHS for a genomic region of interest, and found that the fraction of SNVs |iHS|>2 is often the most powerful iHS summary for detecting selection."
Regarding the iHS results, we did not explore other methods that are often used in selection studies (like Tajima's D and XP-EHH), because we were mainly interested in recent or ongoing selection where the sweep has not yet reached high frequency (because we are mainly interested in selection happening specifically in the MANOLIS cohort, which has only been separated from other European populations for a limited time period) and iHS is more powerful for detecting such selection than Tajima's D [5,6] and XP-EHH[7]. However, we agree that it would be of interest to see if other measures are consistent with selection in *FAM189B.* We have therefore now performed an F_{ST} based analysis of this gene, as suggested by the reviewer, and the results of this analysis are indeed consistent with selection having acted in the MANOLIS cohort. We have added this information both to the results section and the discussion section. Furthermore, we have added the following statement in the discussion to more clearly reflect the nature of the observed evidence (both iHS and F_{ST}):
"However, we caution that although FAM189B is in the top 5% of all genes for both iHS and F_{ST}, it is not an extreme outlier for either, suggesting that it could be a false positive or that the selection has not acted strongly enough or for long enough to leave more than subtle signatures in the haplotype structure and allele frequencies in the gene. It is also possible that selection has acted on several rare alleles making the signature more complex than simple directional selection.".
Finally, we have looked at results reported from the 1000 Genomes Project and searched through selection studies of other populations and have not found *FAM189B* to be reported to be under selection previously. For example, it is not found in dbPSHP, a literature based database of signatures of recent positive selection[8]. We have added this information to the discussion:
"We found FAM189B to contain an elevated fraction of SNVs with |iHS|>2, a potential signature of recent positive selection. Furthermore, FAM189B is in the top 5% of all genes in terms of population differentiation (F_{ST}) between the MANOLIS cohort and the 1000 genome CEU sample, which is consistent with selection having happened in the MANOLIS cohort. This is particularly interesting in the context of this population, which has a high animal fat content diet[9], and for which loss of function variants in APOC3 have risen in frequency compared to the general population and confer a cardioprotective effect[10,11]. For the same reason, it is interesting to note that FAM189B has not previously been reported to be under selection in other populations[8]. "

8. I find the discussion on selection rather weak and the article might benefit from a longer discussion of the population genetics of this cohort (although this might have been done elsewhere; if that is the case, please summarize in the introduction).

Thank you for this suggestion. In addition to the changes outlined in response to the previous comment, we have also added the following in the Introduction:
"The population genetics characteristics of MANOLIS have been studied, and indicate an effective population size of Ne=6,242 and an approximate time of divergence of 1,100 years from the general Greek population[12,13]".

References

1. Gilly, A. *et al.* Very low-depth sequencing in a founder population identifies a cardioprotective APOC3 signal missed by genome-wide imputation. *Hum Mol Genet* **25**, 2360-2365 (2016).
2. TG and HDL Working Group of the Exome Sequencing Project, N.H.L. *et al.* Loss-of-function mutations in APOC3, triglycerides, and coronary disease. *N Engl J Med* **371**, 22-31 (2014).

3. Li, A.H. *et al.* Analysis of loss-of-function variants and 20 risk factor phenotypes in 8,554 individuals identifies loci influencing chronic disease. *Nat Genet* **47**, 640-2 (2015).
4. Moore, C. *et al.* The INTERVAL trial to determine whether intervals between blood donations can be safely and acceptably decreased to optimise blood supply: study protocol for a randomised controlled trial. *Trials* **15**, 363 (2014).
5. Tajima, F. Statistical method for testing the neutral mutation hypothesis by DNA polymorphism. *Genetics* **123**, 585-95 (1989).
6. Voight, B.F., Kudaravalli, S., Wen, X. & Pritchard, J.K. A map of recent positive selection in the human genome. *PLoS Biol* **4**, e72 (2006).
7. Pickrell, J.K. *et al.* Signals of recent positive selection in a worldwide sample of human populations. *Genome Res* **19**, 826-37 (2009).
8. Li, M.J. *et al.* dbPSHP: a database of recent positive selection across human populations. *Nucleic Acids Res* **42**, D910-6 (2014).
9. Farmaki, A.E. *et al.* The mountainous Cretan dietary patterns and their relationship with cardiovascular risk factors: the Hellenic Isolated Cohorts MANOLIS study. *Public Health Nutr* **20**, 1063-1074 (2017).
10. Tachmazidou, I. *et al.* A rare functional cardioprotective APOC3 variant has risen in frequency in distinct population isolates. *Nat Commun* **4**, 2872 (2013).
11. Pollin, T.I. *et al.* A null mutation in human APOC3 confers a favorable plasma lipid profile and apparent cardioprotection. *Science* **322**, 1702-5 (2008).
12. Xue, Y. *et al.* Enrichment of low-frequency functional variants revealed by whole-genome sequencing of multiple isolated European populations. *Nat Commun* **8**, 15927 (2017).
13. Panoutsopoulou, K. *et al.* Genetic characterization of Greek population isolates reveals strong genetic drift at missense and trait-associated variants. *Nat Commun* **5**, 5345 (2014).

Reviewer #2 (Remarks to the Author):

The authors have appropriately addressed my comments.
