# Supplementary material for: Cohort-wide deep whole genome sequencing and the allelic architecture of complex traits
Source: Nat Commun. 2018 Nov 7;9:4674. doi: 10.1038/s41467-018-07070-8 (PMC6220258; doi:10.1038/s41467-018-07070-8)
Supplement: Supplementary file 1 — peer review file [file 41467_2018_7070_MOESM1_ESM.pdf]

Description of Additional Supplementary Files

File Name: Supplementary Data 1
Description: Variants included in the burdens of the selected associations.

File Name: Supplementary Data 2
Description: Variants included in the burdens of the selected associations in the replication Cohort.
